# Pre‐Existing Diabetes Alters Pulmonary Inflammatory Gene Expression Priming for Injury

**DOI:** 10.1096/fj.202500816R

**Published:** 2025-07-14

**Authors:** Abdulaziz H. Alanazi, Mohamed S. Selim, Fang Liu, Duo Zhang, S. Priya Narayanan, Payaningal R. Somanath

**Affiliations:** ^1^ Clinical and Experimental Therapeutics University of Georgia Augusta Georgia USA; ^2^ Charlie Norwood VA Medical Center Augusta Georgia USA; ^3^ Department of Clinical Practice, College of Pharmacy Northern Border University Rafha Saudi Arabia

**Keywords:** acute lung injury, cytokines, diabetes mellitus, inflammation, RNA‐seq, sepsis

## Abstract

Diabetes mellitus (DM) is a systemic disease known for its cardiovascular complications, but its impact on pulmonary health remains underexplored. We aimed to determine how pre‐existing DM influences lung inflammation and susceptibility to acute lung injury (ALI). RNA sequencing was performed on lung tissues from streptozotocin‐induced DM and non‐DM mouse lungs, followed by gene enrichment and bioinformatics analysis. Lung inflammation and injury were assessed in a lipopolysaccharide‐induced sepsis model using Wet/Dry lung weight ratios, histopathology, RT‐qPCR, and cytokine profiling. Transcriptomic analysis revealed that DM lungs exhibited upregulated inflammatory pathways and signs of compromised endothelial barrier integrity. While LPS exposure induced lung inflammation, no additive or synergistic effect of DM and LPS was observed in exacerbating lung injury. However, DM alone was associated with increased expression of inflammatory cytokines (TNF‐α, IL‐1β, MCP‐1, and CXCL‐1), greater fluid accumulation, and structural lung changes indicative of enhanced baseline susceptibility to ALI. These findings underscore the impact of DM on priming the lung for inflammation and injury and suggest that targeting DM‐associated molecular pathways may help mitigate pulmonary complications in diabetic individuals.

## Graphical abstract

Diabetes mellitus (DM) is linked to cardiovascular complications, but its effects on lung health are less understood. Using a streptozotocin‐induced DM mouse model, we found that DM alone upregulated inflammatory pathways, disrupted endothelial integrity, and increased cytokine expression and lung fluid accumulation. While LPS‐induced sepsis triggered inflammation, no additive effect with DM was observed. These findings suggest that DM primes the lung for injury and highlight the need to target DM‐associated pathways to reduce pulmonary complications.

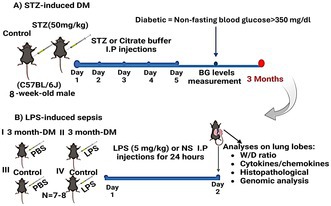

**Abbreviations**
AGER: advanced glycosylation end‐product receptor
ALI: acute lung injury
Angptl4: angiopoietin‐like 4
ARDS: acute respiratory distress syndrome
Cldn5: claudin 5
COPD: chronic obstructive pulmonary disease
CXCL1: C‐X‐C motif chemokine ligand 1
DEGs: differentially expressed genes
DM: diabetes mellitus
GO: gene ontology
GSEA: gene set enrichment analysis
ICU: intensive care unit
KEGG: Kyoto Encyclopedia of Genes and Genomes
LPS: lipopolysaccharide
Ly6c1: lymphocyte antigen 6 complex
MCP‐1: monocyte chemoattractant protein‐1
PCA: principal component analysis
PI3K: phosphatidylinositol 3′‐kinase
Slfn4: schlafen 4
STZ: streptozotocin

## 1 Introduction

Diabetes mellitus (DM) is an intricate systemic disease marked by chronic hyperglycemia, usually associated with inflammation and oxidative stress [1]. It is one of the most common and challenging chronic conditions, significantly burdening individuals and economies globally [2]. According to the International Diabetes Federation, the incidence of DM is increasing at a concerning annual rate, with a total of 536.6 million cases reported in 2021, and is anticipated to reach 783.2 million in 2045 [3]. Over 90% of the cases are attributed to type‐2 DM, while an estimated 8.4 million individuals had a type‐1 DM diagnosis worldwide in 2021 [3]. DM is determined to be the most common co‐morbidity among critically ill patients; about 40% of intensive care unit (ICU) subjects have pre‐existing DM, including those suffering from respiratory disorders like acute respiratory distress syndrome (ARDS) [4].

Morbidity and mortality linked to patients with DM are thought to be a result of endothelial dysfunction, which in turn leads to vascular complications, including retinopathy, nephropathy, neuropathy, accelerated atherosclerosis, and cerebrovascular accidents [5]. The pathophysiology and molecular mechanisms underlying the complications have largely been examined [6]. Although the lungs have a large capillary network, little attention has been given to the lung as a target organ for DM, leading to inadequate understanding of the potential pulmonary complications of DM. Despite the presence of numerous observational studies, the literature reports diverse and often inconsistent findings regarding the association between DM and lung disorders [7, 8, 9, 10, 11]. Among critically ill patients with respiratory failure, acute respiratory distress syndrome (ARDS) is a severe and common complication, with a high mortality rate of 30% to 40% [12, 13].

While DM is a risk factor contributing to adverse outcomes in patients with respiratory diseases, some reports are contradictory [7, 14]. In a clinical study, we previously scrutinized the relationship between DM and ARDS [15], revealing DM as a potential risk factor significantly attributed to unfavorable outcomes involving high death and ICU rates in ARDS patients compared to ARDS subjects with no history of DM [15]. Considering the importance of our robust clinical findings and the critical role of combinatorial studies in validating such a positive association, we expanded our research to thoroughly investigate DM lungs in a well‐designed streptozotocin (STZ)‐induced mouse model. Consequently, this current study evaluated the consequence of DM on lung health in mice, characterized the associated molecular mechanisms involving RNA sequencing, bioinformatics, and biochemical analysis, and investigated the impact of pre‐existing DM on the lipopolysaccharide (LPS)‐induced sepsis model in mice.

## 2 Methods

### 2.1 Mice

All the animal studies were carried out in the Charlie Norwood Veterans Affairs Medical Center, Augusta, and approved by the Institutional Animal Care and Use Committee (Animal Component of Research Protocol # 1604232 approved on 01/28/2023). Eight‐week‐old male C57BL/6J mice with body weights around 23 g were purchased from the Jackson Laboratory (Bar Harbor, ME, USA). For the animal studies, all mice were allocated to experimental groups without randomization. Each set of experiments was conducted simultaneously to minimize variability.

### 2.2 STZ‐Induced DM in Mice

**FIGURE 1:**
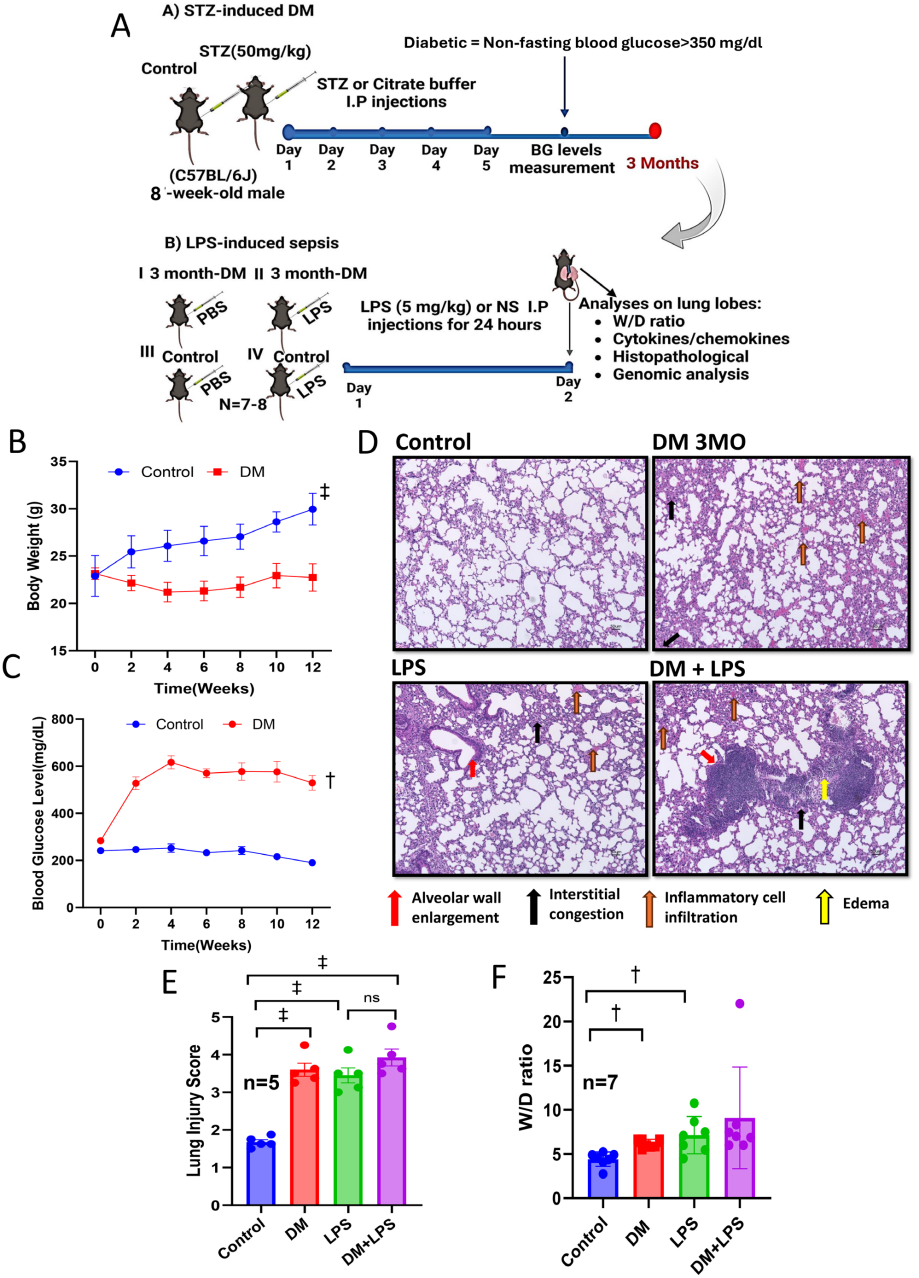
Streptozotocin (STZ)‐induced diabetes compromises lung health and primes for lung injury in mice. (A) A flow chart of the experimental protocol. (B) Body weight measurements (once every 2 weeks) in STZ‐induced DM mice compared to non‐DM mice (*n* = 7). (C) Blood glucose levels (once every 2 weeks) in STZ‐induced DM mice compared to non‐DM mice (*n* = 7). (D) Representative images of H&E staining demonstrating pathological alterations in lipopolysaccharide (LPS)‐treated, 3‐month DM, and 3‐month DM mice treated with LPS, compared to control (vehicle‐treated) non‐DM mice (*n* = 7). (E) Bar graph showing quantification (blinded analysis) of lung injury scores in LPS‐treated, 3‐month DM, and 3‐month DM mice treated with LPS (i.p.) 24 h prior to 3 months, compared to control (vehicle‐treated) non‐DM mice (*n* = 5). (F) Bar graph showing wet/dry ratio of lung tissues in LPS‐treated, 3‐month DM, and 3‐month DM mice treated with LPS, compared to control (vehicle‐treated) non‐DM mice (*n* = 7). ^†^ *p* < 0.01 (glucose levels), ^‡^ *p* < 0.001 (body weight).Streptozotocin (STZ)‐induced diabetes compromises lung health and primes for lung injury in mice. (A) A flow chart of the experimental protocol. (B) Body weight measurements (once every 2 weeks) in STZ‐induced DM mice compared to non‐DM mice (*n* = 7). (C) Blood glucose levels (once every 2 weeks) in STZ‐induced DM mice compared to non‐DM mice (*n* = 7). (D) Representative images of H&E staining demonstrating pathological alterations in lipopolysaccharide (LPS)‐treated, 3‐month DM, and 3‐month DM mice treated with LPS, compared to control (vehicle‐treated) non‐DM mice (*n* = 7). (E) Bar graph showing quantification (blinded analysis) of lung injury scores in LPS‐treated, 3‐month DM, and 3‐month DM mice treated with LPS (i.p.) 24 h prior to 3 months, compared to control (vehicle‐treated) non‐DM mice (*n* = 5). (F) Bar graph showing wet/dry ratio of lung tissues in LPS‐treated, 3‐month DM, and 3‐month DM mice treated with LPS, compared to control (vehicle‐treated) non‐DM mice (*n* = 7). ^†^ *p* < 0.01 (glucose levels), ^‡^ *p* < 0.001 (body weight).

STZ‐induced type 1 DM, a well‐established DM model, was utilized in our study [16]. This model was selected for its reproducibility, well‐characterized progression of hyperglycemia, and consistent systemic inflammation, making it ideal for assessing the impact of chronic diabetes on lung responses within a defined experimental timeframe. Male C57BL/6 mice were subjected to intraperitoneal STZ injections of 50 mg/kg dissolved in citrate buffer once a day [17] for five consecutive days, with control groups receiving only citrate buffer injections. A drop of blood was collected via the tail vein to measure blood glucose levels at least twice a month using a glucometer (Alpha TRAK2 blood glucose monitoring system, Fisher Scientific, Pittsburgh, PA, USA). Mice whose blood glucose levels were above 350 mg/dL (non‐fasting) were included in the DM cohort. Mouse body weights were recorded biweekly during the study period. Twelve weeks after STZ administration, mice were confirmed as diabetic, assigned to experimental groups, euthanized, and their lungs collected for analysis. To assess the impact of pre‐existing DM on the severity of acute lung injury (ALI), we utilized a clinically relevant model of sepsis‐induced ALI. For this experiment, 12‐week DM mice were intraperitoneally challenged with LPS at a dose of 5 mg/kg, and non‐DM mice who served as positive controls received LPS only [17]. After 24 h of LPS treatment, mice were sacrificed, and lungs were extracted for examination. The final group allocation of our experiment was as follows: (1) Control, (2) DM, (3) LPS‐treated controls (LPS), and (4) LPS‐treated DM mice (DM + LPS). A flow chart of the experimental protocol is illustrated in Figure 1A.

### 2.3 Lung Wet/Dry Weight Ratio Analysis

To measure lung edema, lungs from all the experimental mice were retrieved, and the upper right lobes were freshly weighed before incubating them in an oven for 72 h at 80°C. Following that, dry weights were measured, and final wet/dry ratios were calculated to estimate the water content in the lungs.

### 2.4 Histopathological Analysis and Lung Injury Scoring

To examine and compare the pathological changes associated with DM or DM with sepsis (DM + LPS) mice compared to their respective controls, lungs were collected, immediately fixed in 4% paraformaldehyde, and sectioned before being stained with H&E. Following that, lung tissues were assessed using microscopy, and lung injury was blindly scored based on already developed criteria [18]. Briefly, a scale of 1–5 was utilized to score all the samples, with 1 being normal to minimal damage lung, whereas 5 indicated critical lung injury evidenced by diffuse (occurs in more than 50% of lung section) consolidation concurrent with inflammatory cell infiltration.

### 2.5 qRT‐PCR Analysis of Inflammatory Cytokines in Lung Tissues

Lung RNA analysis was performed as mentioned previously [18]. Briefly, RNA was isolated from mice lung tissues utilizing a miRNeasy mini kit (Qiagen). The quality of the RNA was checked via a Nanodrop 2000 spectrophotometer (Thermo Scientific). Around 1000 ng of the total RNA was used for Complementary DNA (cDNA) synthesis employing a High‐Capacity cDNA Reverse Transcript Kit (Applied Biosystems, Waltham, MA, USA). Quantitative real‐time PCR (qRT‐PCR) was conducted using Power SYBR Green Master Mix (Applied Biosystems) in the StepOnePlus Real‐Time PCR System (Applied Biosystems). All mouse primers included in the analysis are listed in Table S1.

### 2.6 RNA‐Sequencing and Bioinformatics Analysis

Lung tissues from DM and non‐DM (control) mice were taken out, RNA was isolated, and then quality control tests were performed to ensure these samples were pure and integrated using NanoDrop 2000 (Thermo Scientific) and the Agilent 2100 bioanalyzer, respectively. Total RNAs that passed the quality control test were further processed for cDNA library preparation employing Illumina NovaSeq 6000 and X‐Plus Sequencing Platform with PE150 strategy, followed by bioinformatics analysis. All differentially expressed genes (DEG) among DM versus non‐DM arms were subjected to comprehensive gene set enrichment analysis (GSEA) and gene ontology (GO). Several databases for pathway enrichment analysis, such as Kyoto Encyclopedia of Genes and Genomes (KEGG) and Reactome, were included in our analysis to detect signaling pathways related to DEGs. R program and SRplot were used to carry out gene distribution analysis, incorporating PCA, volcano plot, and heatmaps [19]. A Venn diagram was created to show genes that were uniquely expressed in each group, along with upregulated and down‐regulated genes in the DM group. While the PCA was used to demonstrate that the DM group did not overlap with the control group, volcano plots and heatmaps were performed to reveal the top DEGs in DM lung tissues. The violin plots were created using GraphPad software. The interactions between DEGs were assessed utilizing STRING [20].

### 2.7 Statistical Analysis

All the data are depicted as means ± SEM, and the “*n*” value for each dataset indicates the sample size used for that experiment. All the data were assessed by parametric testing using Student's unpaired *t*‐test or one‐way ANOVA. In bioinformatic analysis, DEGs with fold change values > 0.5 or < 0.5 are deemed to be upregulated and downregulated, respectively. In all the analyses, a *p*‐value < 0.05 is considered statistically significant.

## 3 Results

### 3.1 STZ‐Induced Diabetes Impaired Lung Health Without an Additive Effect in LPS‐Induced Sepsis in Mice

To confirm the successful induction and maintenance of diabetes by STZ throughout the experiment, blood glucose levels were measured bi‐weekly. We achieved 100% success in inducing diabetes in the STZ‐treated mice without any mortality, with all animals meeting the blood glucose criteria for DM confirmation. As expected, DM mice showed a significant increase in blood glucose levels, ranging from 350 to 600 mg/dL (Figure 1C). Additionally, body weights were regularly monitored, revealing a modest but significant weight reduction in DM mice compared to control groups (Figure 1B).

To assess the impact of DM on the lungs and its influence as a comorbid condition in sepsis‐induced ALI, we performed histopathological examinations on lung sections from all experimental groups. Our analysis revealed a significant increase in lung injury scores in DM mice compared to non‐DM mice (Figure 1D,E). Histological evaluation showed inflammatory cell infiltration and mild interstitial congestion in the lungs of DM mice (Figure 1D,E). When assessing the effect of pre‐existing DM in septic mice, we observed that lung tissues from DM mice with sepsis exhibited increased inflammatory cell infiltration, thickened alveolar walls, and more pronounced interstitial congestion and edema compared to non‐DM sepsis or DM‐only mice (DM + LPS vs. LPS or DM). Although these observations suggested more extensive injury, the lung injury scores in DM + LPS mice were not significantly higher than those in LPS‐only mice, indicating no additive effect of DM and LPS in worsening lung injury (Figure 1D,E). Our H&E staining data revealed substantial histopathological changes in DM lung tissues. However, these changes were not significantly exacerbated by the addition of LPS, suggesting that while DM impairs lung health, it does not necessarily intensify sepsis‐induced injury.

Since pulmonary edema is a key indicator of ALI, we assessed lung water content using the wet‐to‐dry ratio to further evaluate the impact of DM alone and as a comorbid condition in sepsis‐induced ALI. Our results showed a significantly higher water content in DM mouse lungs compared to non‐DM lungs, suggesting that DM increases capillary permeability, facilitating fluid leakage into the interstitial and air spaces (Figure 1F). As expected, sepsis mice (LPS) exhibited significantly increased pulmonary edema compared to controls (Figure 1F). Although lung fluid content appeared modestly higher in DM + LPS mice compared to either condition alone, this difference did not reach statistical significance, further supporting the absence of an additive effect (Figure 1F).

### 3.2 RNA Sequencing Analysis of DM Lungs Shows Activation of Pathways Involved in Inflammation and Vascular Injury

**FIGURE 2:**
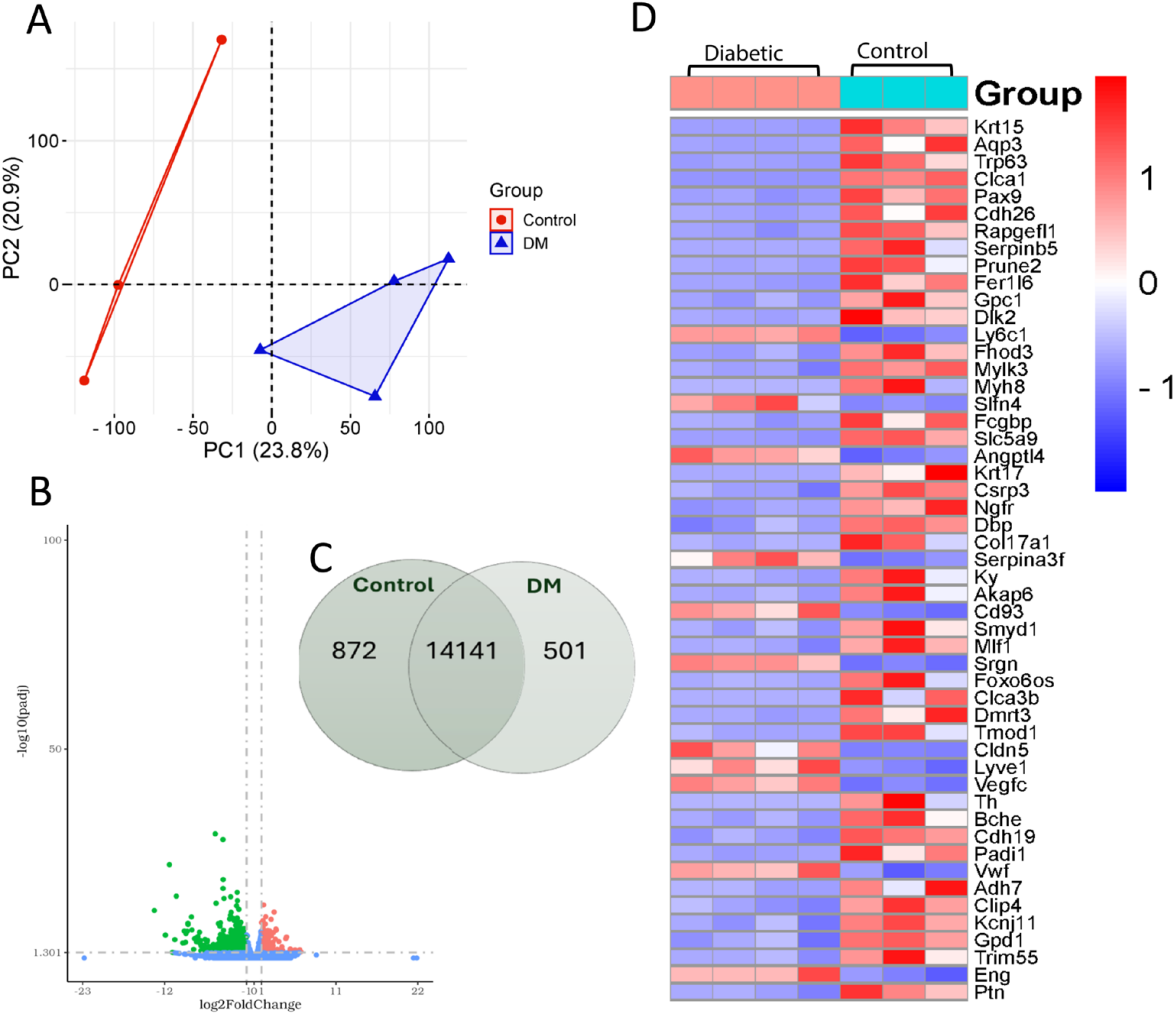
RNA sequencing analysis showing differential gene expression in DM mouse lungs compared to non‐DM mouse lungs. (A) Principal component analysis (PCA) reveals a significant separation of gene expressions between DM and non‐DM lung samples. (B) The volcano plot shows differentially expressed genes (DEGs), with genes upregulated in DM lung tissues appearing on the right and those downregulated in DM compared to non‐DM tissues on the left. (C) Venn diagram showing the number of identified genes between the two groups. (D) The heatmap displays the top DEGs in the DM and non‐DM lung tissues (*n* = 3–4).

Given the intriguing observations in diabetic lungs, we conducted RNA‐seq analysis to explore the transcriptional effects of DM on lung tissue compared to non‐DM mouse lungs. The principal component analysis (PCA) revealed a clear segregation between DM and non‐DM lung samples, suggesting that DM is associated with a distinct gene expression signature (Figure 2A). Furthermore, the transcriptomic analysis identified 14 141 genes commonly expressed in both DM and non‐DM lungs, with 501 genes exclusively detected in diabetic lungs (Figure 2B,C). Differential gene expression analysis revealed significant alterations, with 203 genes upregulated and 671 genes downregulated in diabetic lungs compared to controls, as illustrated in the volcano plot (Figure 2B). To further highlight the most DEGs in diabetic lungs, we generated a heatmap displaying the top upregulated and downregulated genes (Figure 2D).

**FIGURE 3:**
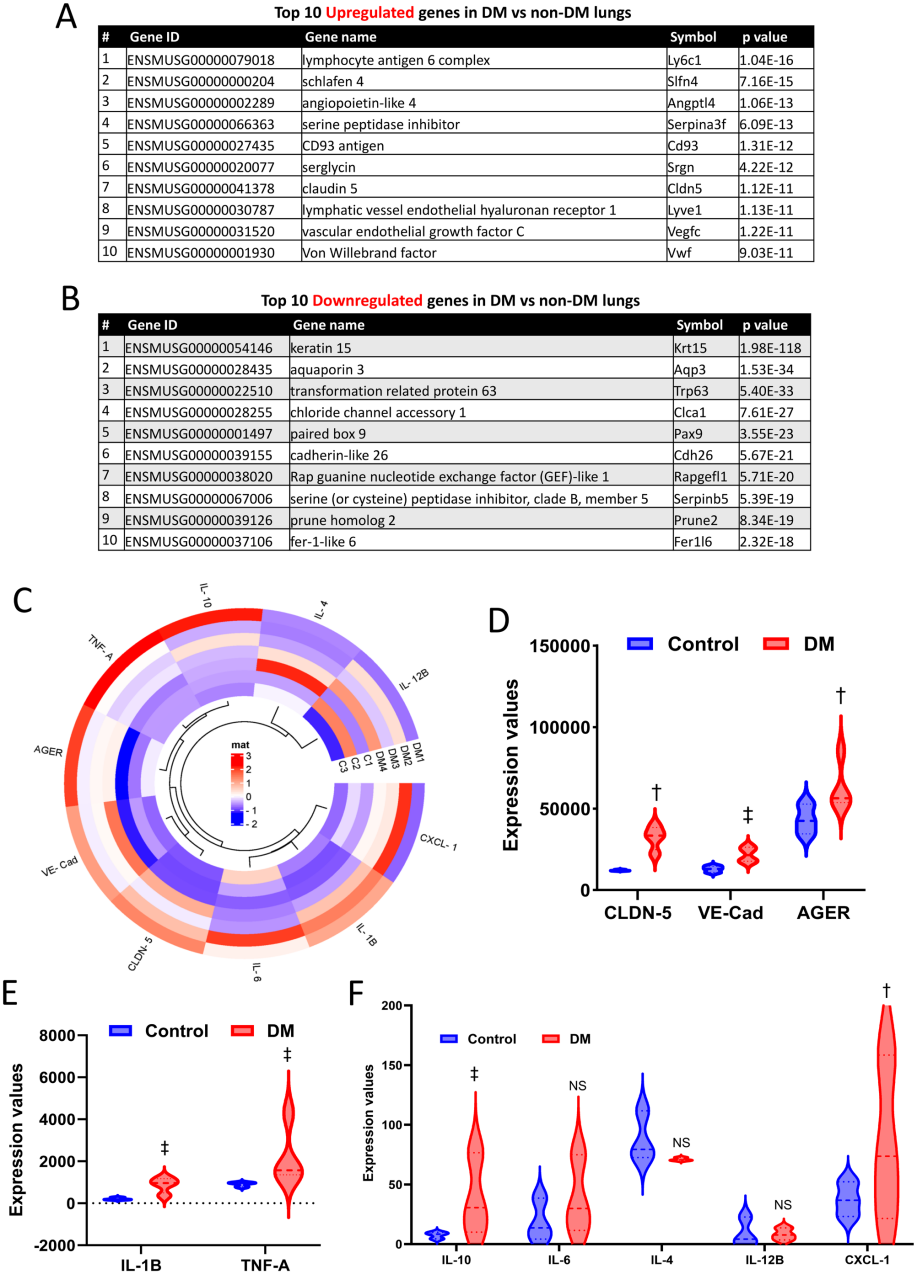
The top DEGs in DM mouse lungs suggest inflammation and endothelial cell abnormalities. (A, B) List of the top upregulated and downregulated genes in DM lung tissues compared to non‐DM lung tissues, respectively. (C) Donut heatmap representing the expression of the inflammatory and endothelial genes in DM lungs compared to non‐DM lungs. (D–F) Violin plots demonstrating expression changes in Cldn5, VE‐Cadherin, AGER, IL1β, TNF‐α, IL‐10, IL‐6, IL‐4, IL‐12B, and Cxcl‐1 in DM compared to non‐DM lungs (*n* = 3–4). NS, not significant; C1–C3 are controls 1–3; DM1–4 are diabetic samples 1–4. ^†^ *p* < 0.01, ^‡^ *p* < 0.001.

Following our initial genomic profiling, we conducted a detailed analysis of DEGs in DM lungs compared to non‐DM lungs. Our findings revealed a significant upregulation of genes involved in inflammatory responses (Figure 3A). Notably, lymphocyte antigen 6 complex (Ly6c1), predominantly expressed in the lung, was significantly elevated in DM lung samples. Additionally, schlafen 4 (Slfn4), a vascular inflammatory modulator, and angiopoietin‐like 4 (Angptl4), associated with chronic low‐grade inflammation, were markedly increased, indicating increased immune activation. In DM lungs, genes regulating endothelial and epithelial barrier integrity, such as claudin 5 (Cldn5) and VE‐Cadherin, were upregulated, suggesting DM‐induced vascular dysfunction. Conversely, several genes showed reduced expression in the DM group compared to controls (Figure 3B). For instance, Aqp3, which regulates water movement and maintains fluid homeostasis in the lungs, was significantly downregulated in diabetic lung tissues. Moreover, our RNA‐seq data revealed alterations in several pro‐ and anti‐inflammatory genes, including TNF‐α, IL‐1β, IL‐6, CXCL‐1, IL‐10, and IL‐4, as depicted in donut heatmaps and violin plots (Figure 3C,E,F). Additionally, the receptor for advanced glycation end products (AGER), a key contributor to DM‐related complications, was significantly increased in DM lungs (Figure 3C,D). Collectively, these findings provide strong evidence of inflammation and edema in lungs affected by DM.

### 3.3 GO, KEGG and Reactome Analysis Reveal Activation of Metabolic and Inflammatory Pathways in DM Lungs

**FIGURE 4:**
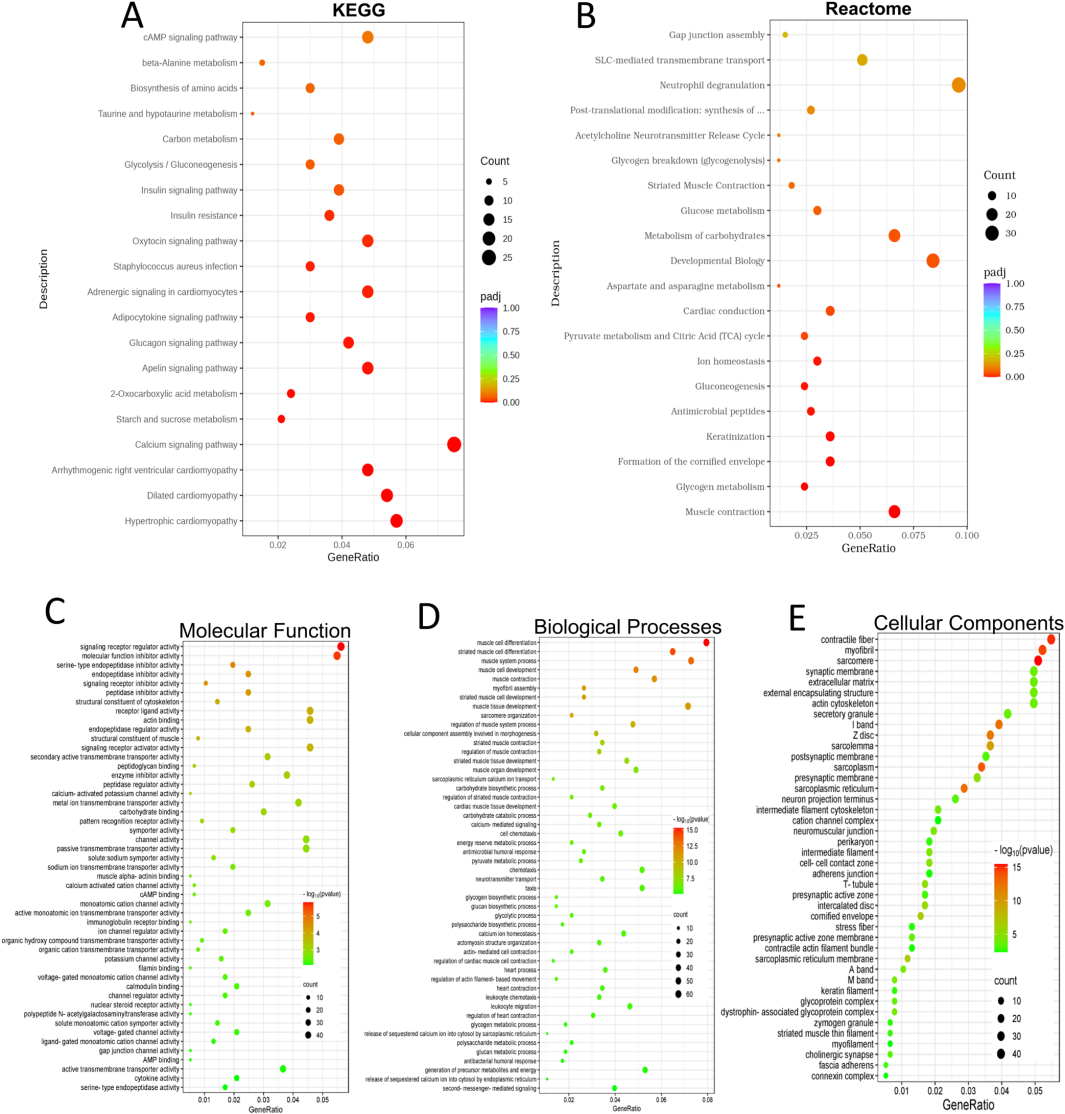
Gene enrichment analysis shows activation of pathways regulating inflammation, metabolism, and vascular abnormalities in DM compared to non‐DM lungs. (A, B) KEGG and Reactome pathway analysis plots demonstrating top altered molecular pathways in the DM lungs. (C–E) GO analysis shows DEGs involved in molecular function, biological processes, and cellular components, respectively.

To identify deregulated signaling pathways associated with DEGs in DM mouse lungs, we conducted a comprehensive pathway analysis using multiple databases, including KEGG and Reactome. KEGG analysis revealed significant alterations in metabolic pathways, such as beta‐alanine metabolism, Apelin signaling, and insulin signaling, including its resistance mechanisms. Additionally, pro‐inflammatory pathways, such as adipocytokine signaling and immune responses, including *Staphylococcus aureus* infection, were significantly involved in DM lungs (Figure 4A). Consistent with KEGG findings, Reactome pathway analysis further confirmed the involvement of DEGs in metabolic and inflammatory pathways. Specifically, dysregulated metabolic pathways were linked to ion homeostasis, glucose metabolism, and gluconeogenesis, while immune‐related pathways, such as neutrophil degranulation and antimicrobial peptide activity, were significantly affected in DM lungs. Notably, DEGs in DM lungs also contributed to the disruption of gap junction assembly (Figure 4B). Collectively, these findings indicate that DM lungs undergo significant metabolic disturbances, immune activation, and gap junction dysregulation. Given these observations, a more detailed analysis of these altered pathways and their associated DEGs is essential to uncover potential therapeutic strategies for mitigating DM‐induced lung injury.

**FIGURE 5:**
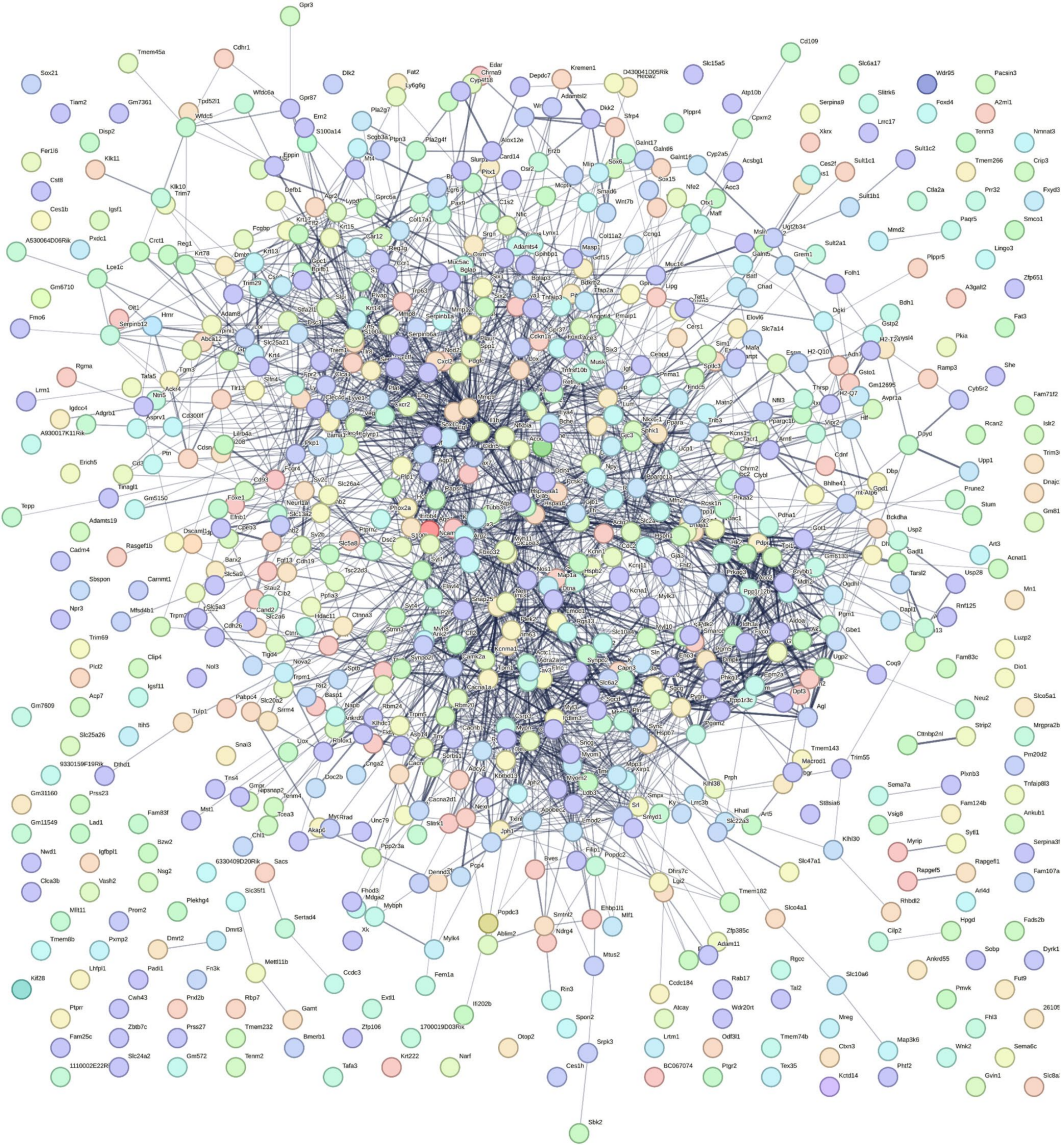
STRING network analysis illustrating the interaction among all the identified DEGs in DM lungs.

Next, we performed GO and GSEA. GO analysis revealed that the top molecular functions (MFs) of altered genes were associated with signaling receptor regulation, peptidase inhibitor/regulator activity, ion channel regulation, gap junction channel function, and cytokine activity, as shown in (Figure 4C). Cellular component (CC) analysis identified key structural and functional elements, including contractile fibers, myofibers, cell–cell contact zones, glycoprotein complexes, and adherens junctions (Figure 4E). Meanwhile, enriched biological processes (BPs) of DEGs were linked to myofiber assembly, antimicrobial humoral responses, glycogen metabolism, and leukocyte chemotaxis/migration (Figure 4D). A comprehensive list of CCs and BPs is presented in Figure 4D,E. Additionally, we explored interactions among DEGs, revealing multiple gene–gene interactions in DM lungs (Figure 5).

**FIGURE 6:**
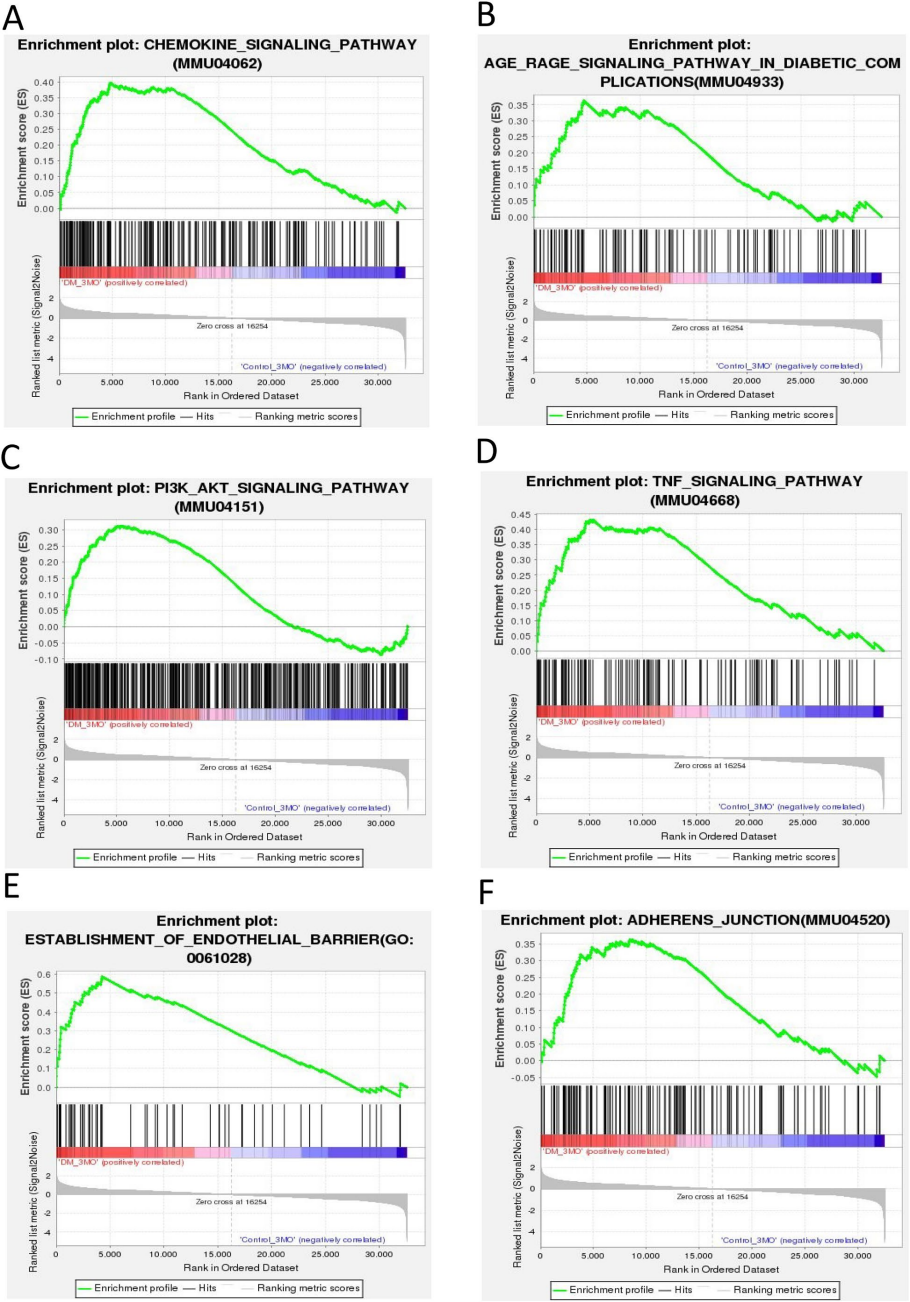
Gene set enrichment analysis (GSEA) of DEGs in the tissue of DM lungs. (A–F) Enrichment plots profiling the positive correlation between altered genes and the molecular pathways in DM lungs including Chemokine signaling (A), AGER signaling (B), PI3 Kinase‐Akt signaling (C), TNF‐α signaling (D), endothelial‐barrier regulation (E), and Adherens junction regulation (F).

GSEA revealed the enrichment of several inflammatory‐related signaling pathways, including chemokine signaling, TNF‐α, phosphatidylinositol 3′‐kinase (PI3K)‐AKT, and AGE‐AGER pathways, in the lungs of DM mice compared to non‐DM mice (Figure 6A–D). Interestingly, genes involved in the development of endothelial barriers, adherens junctions, and vasculature were notably modulated in DM lungs (Figure 6E,F). Overall, these findings highlight an overactive immune response and disrupted metabolic processes in diabetic lung tissue, both of which could contribute to the development of vascular dysfunction.

### 3.4 DM Mouse Lungs Generated Increased Inflammatory Cytokines, but Sepsis Did Not Significantly Worsen This Response

**FIGURE 7:**
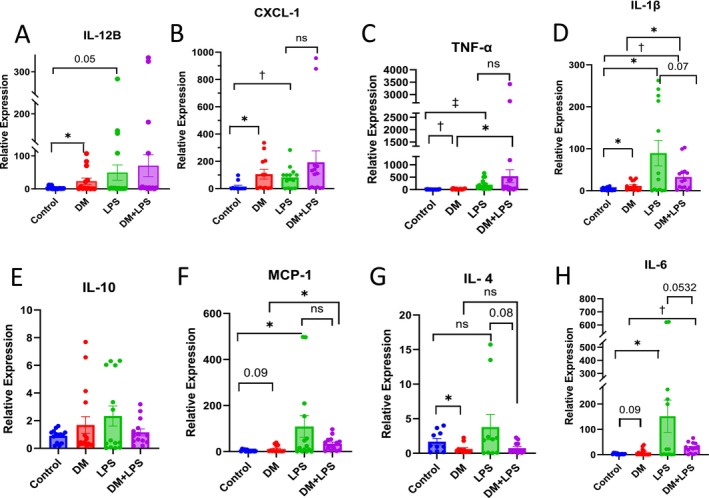
Analysis of lung homogenates showing increased expression of cytokines in DM mouse lungs (3 months after STZ administration). (A–H) Bar graphs showing changes in mRNA levels of IL‐12B (A), CXCL‐1 (B), TNF‐α (C), IL‐1β (D), IL‐10 (E), MCP‐1 (F), IL‐4 (G), and IL‐6 (H) in lung tissues with DM compared to non‐DM lungs and LPS‐treated (24 h prior to 3 months post‐STZ adninstration) DM lungs compared to vehicle‐treated DM mice (*n* = 7). Data are shown as mean ± SEM. *p < 0.05, ^†^ *p* < 0.01, ^‡^ *p* < 0.001.

Given the significant pathological changes in the lungs of DM mice and the exacerbation of ALI by pre‐existing DM in septic mice, we aimed to assess the degree of inflammation in lung homogenates across experimental groups. Using a panel of inflammatory cytokines and chemokines, including TNF‐α, IL‐1β, IL‐6, IL‐10, IL‐4, IL‐12B, monocyte chemoattractant protein‐1 (MCP‐1), and CXCL‐1, we first compared the inflammatory levels between DM and non‐DM mouse lungs. We found that DM mouse lungs had significantly higher levels of pro‐inflammatory markers, such as TNF‐α, IL‐1β, MCP‐1, and CXCL‐1, compared to non‐DM lungs. Conversely, anti‐inflammatory cytokines like IL‐4 were significantly downregulated in DM lungs (Figure 7A–H). These results strongly suggest that the lung environment is inflamed and adversely affected by DM.

Next, we evaluated whether pre‐existing DM significantly amplified the inflammatory response in sepsis mice. While there were trends toward elevated cytokine levels in the DM + LPS group compared to the LPS‐only group, these differences did not reach statistical significance, indicating no clear additive effect of DM on sepsis‐induced inflammation. Significantly higher expressions of pro‐inflammatory markers, such as IL‐12B, CXCL‐1, and TNF‐α, were not observed in septic DM mouse lungs compared to sepsis mice without DM (Figure 7A–H). Interestingly, IL‐1β was significantly higher in the LPS‐only group compared to the DM + LPS group (Figure 7D). Anti‐inflammatory cytokines, such as IL‐4 and IL‐10, showed a strong trend toward downregulation in DM + LPS mice compared to the LPS‐only group (Figure 7E–G).

When comparing DM mouse lungs with and without sepsis (DM + LPS vs. DM), we observed that most pro‐inflammatory markers, including TNF‐α, IL‐1β, MCP‐1, and IL‐6, were significantly elevated in both DM and sepsis, but none were significantly higher in the DM + LPS group compared to either of the single arms (Figure 7A–H). While IL‐12B and CXCL‐1 levels were higher in sepsis DM mice compared to DM‐only mice, these differences were not statistically significant. Additionally, anti‐inflammatory cytokines, IL‐4 and IL‐10, tended to be downregulated in DM + LPS mice compared to DM‐only mice (Figure 7A–H).

Overall, our findings suggest that DM promotes a pro‐inflammatory lung environment; however, the presence of sepsis did not significantly exacerbate inflammatory cytokine expression beyond individual effects, indicating no consistent additive inflammatory response in DM + LPS mice compared to sepsis or DM alone.

## 4 Discussion

DM, characterized by persistent hyperglycemia and inflammation, is a widespread and complex chronic disease that significantly affects both individuals and economies worldwide [21]. While vascular complications of DM are well‐documented, pulmonary complications remain inadequately studied, with inconsistent findings [22, 23]. ARDS, a severe and prevalent complication, often leads to respiratory failure in ICU patients [24]. Given that nearly half of ICU patients have DM as a comorbid condition, it is crucial to investigate how DM influences respiratory disorders, particularly diseases with high mortality rates, such as ARDS, which carries a mortality rate of around 40% [24]. Our recent clinical study suggested a positive relationship between DM and ARDS [15], but literature on lung inflammation and the effect of comorbid DM on lung disease remains limited. Therefore, our study aimed to explore how pre‐existing DM affects lung health and exacerbates sepsis‐induced ALI. Our results demonstrate significant alterations in inflammatory cytokine production, injury, and edema in DM mouse lungs compared to controls associated with deregulated metabolic, inflammatory, and vascular pathways. However, the combination of DM and sepsis did not produce a statistically significant additive effect in worsening lung injury or inflammation, suggesting independent but not synergistic effects.

A recent study reported significant immune cell infiltration in the lungs of 33‐week‐old type 2 DM mice, with an increase in polymorphonuclear‐myeloid‐derived suppressor cells (PMN‐MDSCs) [25]. In contrast, DM rat serum analysis showed reduced neutrophil migration, indicating compromised immune function [26]. Our findings in type 1, STZ‐induced DM mice after 3 months align with these results. Despite differences in experimental models, our data support the role of inflammation in DM lungs. Consistent with another report [27], we observed significant upregulation of IL‐6 expression in DM lungs, suggesting that systemic inflammation in DM extends to the lungs.

The relationship between DM and ALI is characterized by diverse and inconsistent findings [22, 23]. While some studies suggest that DM may reduce the risk of ARDS progression without affecting mortality, others report that DM significantly increases the risk of ARDS compared to non‐DM individuals [23]. A large cohort analysis supports the hypothesis that DM elevates the likelihood of ARDS development [28]. These discrepancies might arise from research designs that were not fully controlled or may have had other limitations. Our recent secondary analysis of the Fluid and Catheter Treatment Trial (FACTT) also found a significant association between pre‐existing DM and poor outcomes in ARDS patients [15]. In preclinical studies, hyperglycemia caused significant lung tissue damage in an LPS‐induced sepsis rat model [29]. Similarly, studies on both type 1 (STZ, 4 weeks) [30] and type 2 DM [31] mice infected with SARS‐CoV‐2 found higher inflammation and pulmonary injury compared to non‐DM mice. Aligning with these observations, our RNA sequencing and GSEA analyses identified deregulated TNF‐α, PI3K‐AKT, AGER, and endothelial barrier signaling pathways, and revealed disruptions in metabolic, inflammatory, and vascular networks in DM lungs. However, despite evidence of inflammation and injury, our findings did not show a statistically significant exacerbation of sepsis‐induced ALI in the presence of DM, suggesting no additive effect under the current experimental conditions.

These findings are consistent with a previous study showing that immune system impairment in DM mice increases their susceptibility to sepsis [32]. In our model, septic DM mice exhibited trends toward elevated levels of pro‐inflammatory markers, including IL‐12B, CXCL‐1, and TNF‐α, and reduced IL‐4 levels compared to mice with sepsis or DM alone; however, these differences did not consistently reach statistical significance. Other upregulated genes include Ly6c1, which regulates inflammation and complement activity, and Slfn4, implicated in systemic inflammation and atherosclerosis [33, 34]. Angptl4, a multifunctional cytokine involved in inflammation and endothelial injury [35], with higher serum levels linked to reduced lung function and systemic inflammation in chronic obstructive pulmonary disease (COPD) patients [36], and correlated with disease severity and mortality in ARDS patients [37, 38, 39], was highly upregulated in DM lungs, highlighting its role in lung inflammation. Additionally, upregulation of Serpina family genes is associated with lung disorders like pulmonary fibrosis and COPD [40, 41, 42]. Collectively, these results support a pro‐inflammatory phenotype in DM lungs but do not provide definitive evidence of increased susceptibility to ALI when DM is combined with sepsis.

Pathological assessments of DM lungs compared to non‐DM control lungs suggest potential dysfunction in the pulmonary vascular endothelium. Genes CD93 and claudin‐5, both primarily expressed in endothelial cells, were among the top upregulated genes, suggesting their role in modulating vascular permeability in DM lungs. Increased Cldn5 expression has been shown to impair vascular structure, leading to lung damage due to enhanced permeability and edema [43, 44]. Elevated Cldn5 expression in lung epithelium is associated with increased permeability, promoting edema and contributing to lung damage [45, 46, 47]. Additionally, we previously published that upregulation of Cldn5 is associated with DM retinopathy, a disease marked by retinal vasculature impairment and inflammation [48, 49]. In contrast, genes like keratin‐15 and aquaporin‐3 were significantly downregulated in DM lungs. Keratin‐15 knockout mice exhibited severe lung injury and inflammation in a COPD model [50], and the reduced expression of keratin‐15 in DM lungs may contribute to increased lung vulnerability. Similarly, Aqp3, essential for respiratory function, has been shown that its deficiency negatively impacts lung health [51]. These dysregulated genes could serve as potential prognostic markers or novel therapeutic targets for mitigating DM‐related lung injury.

Although pre‐existing diabetes clearly impaired lung health and induced baseline inflammation, we did not observe a statistically significant additive effect when combined with LPS‐induced sepsis across several outcome measures. This may be due to a “ceiling effect,” where LPS alone triggers maximal or near‐maximal lung injury, limiting the capacity to detect further exacerbation [52]. Additionally, the STZ‐induced diabetes model may represent a chronic, low‐grade inflammatory state that does not acutely synergize with LPS‐triggered inflammation. Compensatory mechanisms, such as activation of anti‐inflammatory pathways or immune adaptation in diabetic mice, may also blunt further injury upon secondary insult [53]. The use of a single time point and biological variability within the LPS model could have obscured transient or subtle differences. ARDS is a heterogeneous syndrome with various triggers, while we investigated i.p. administered LPS‐induced injury, modeling systemic infection, but differences would likely emerge with other models. Alternative models, such as infectious lung injury or ventilator‐induced lung injury, may better reflect progressive or multifactorial pathogenesis and could more effectively reveal the impact of chronic metabolic dysfunction on lung vulnerability. A type 2 DM model might also better demonstrate additive effects, as it more closely mirrors the metabolic, vascular, and inflammatory profiles seen in ARDS patients. Collectively, these findings underscore the complexity of interactions between chronic metabolic disease and acute inflammatory stress, emphasizing the importance of model selection, timing, and sensitive outcome measures in evaluating such interactions.

One limitation of this study is the use of the STZ‐induced type 1 DM mouse model, which may not fully replicate the complexities of human DM, particularly in terms of long‐term disease progression and comorbidities. We utilized an untreated type 1 DM model, which may not fully reflect the clinical presentation of primarily treated type 1 or type 2 DM patients who typically have residual insulin function, distinct metabolic profiles, and are managed with glucose‐lowering therapies. Additionally, while significant inflammation and injury were observed in DM lungs, the temporal dynamics of these changes and the potential reversibility of lung damage with therapeutic interventions were not explored. RNA sequencing, while useful, may overlook some gene interactions or regulatory mechanisms not captured by mRNA expression alone. The reliance on a sepsis model to induce ALI also limits the generalizability of results, as it may not fully represent other types of lung injury seen in patients with DM, such as those caused by infections or environmental factors. Furthermore, the influence of other comorbidities commonly associated with DM, like cardiovascular disease, was not investigated, which could be relevant in understanding the broader impact of DM on lung health. Despite these limitations, our study underscores that DM impairs lung health and promotes inflammation and endothelial dysfunction. However, we did not observe a statistically significant additive effect of DM on sepsis‐induced ALI outcomes, suggesting that while DM creates a primed inflammatory environment, it may not always synergistically exacerbate lung injury in the context of sepsis. Key genes involved in inflammatory, metabolic, and vascular pathways increase the susceptibility of DM lungs to injury, highlighting the need for further research into the mechanisms linking DM to lung complications and potential therapeutic targets.

## Author Contributions

A.H.A., M.S.S., and P.R.S. designed the animal studies, performed, and analyzed all mouse lung tissues. A.H.A. performed the lung wet/dry ratio, compiled the blinded injury analysis, and the bioinformatics analysis of the RNA sequencing data. D.Z. and S.P.N. contributed to study design and data interpretation and critically reviewed the manuscript. All authors approved the final manuscript version. A.H.A. and P.R.S. conceived and designed the study, analyzed the data, wrote the manuscript, and are the guarantors of this work.

## Conflicts of Interest

The authors declare no conflicts of interest.

## Supporting information


Appendix S1.

## Data Availability

All data generated in this study have been included in the main manuscript or Supporting Information. Any relevant raw data will be made available upon request.
