# Supplementary material for: Pre‐Existing Diabetes Alters Pulmonary Inflammatory Gene Expression Priming for Injury
Source: FASEB J. 2025 Jul 14;39(14):e70804. doi: 10.1096/fj.202500816R (PMC12257442; doi:10.1096/fj.202500816R)
Supplement: Supplementary file 1 — Appendix S1. [file FSB2-39-e70804-s001.zip › fsb270804-sup-0001-Figures.pdf]

A

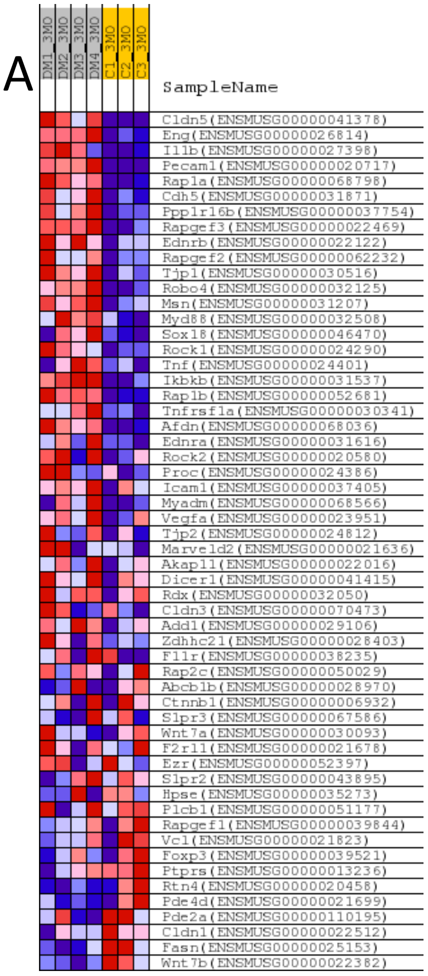

B

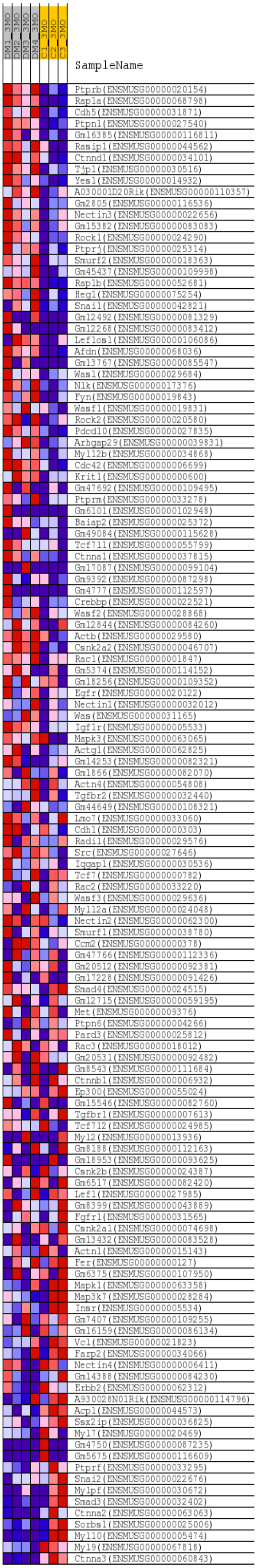

C

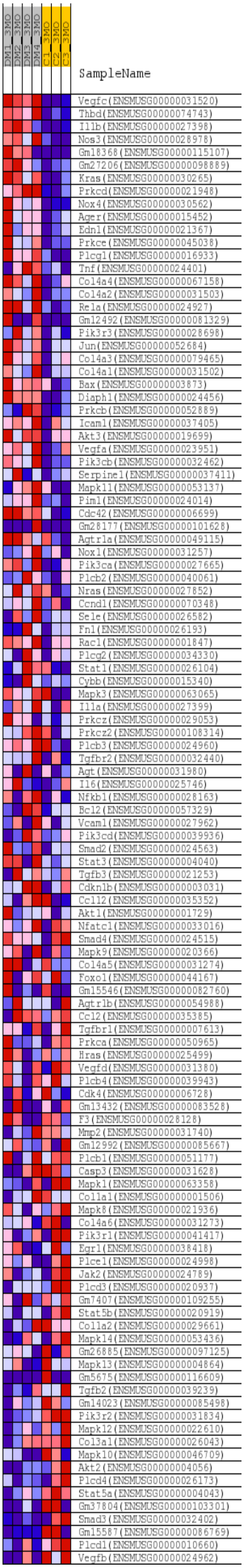

Supplemental Figure 1: Heat maps of DEGs in DM lungs compared to non-DM lungs relating to Chemokine signaling **(a)**, AGER signaling **(b)**, and PI3 Kinase-Akt signaling. Refer Fig. 6 for color coding.

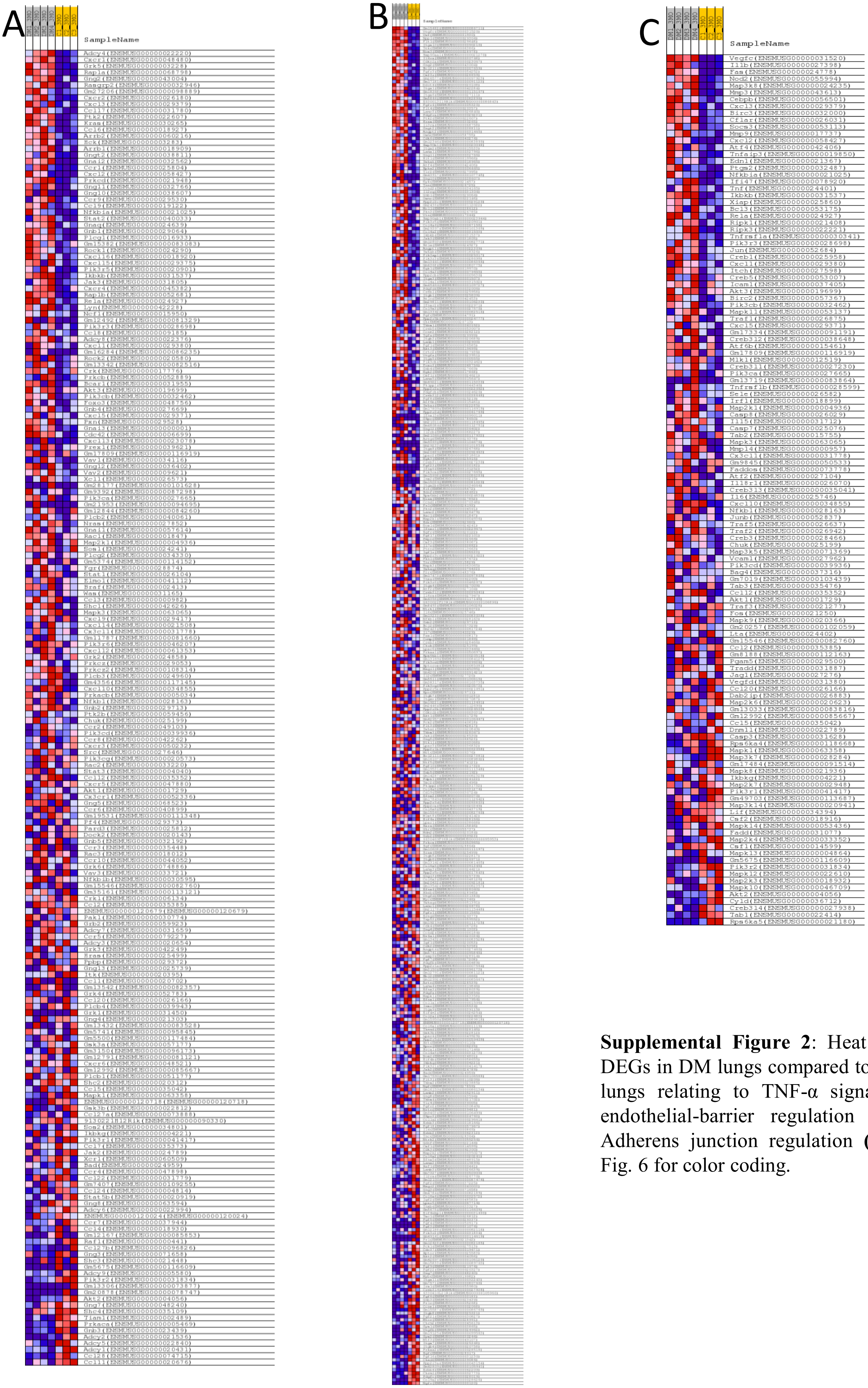

Supplemental Figure 2: Heat maps of DEGs in DM lungs compared to non-DM lungs relating to TNF-α signaling **(a)**, endothelial-barrier regulation **(b)**, and Adherens junction regulation **(c)**. Refer Fig. 6 for color coding.
