# Supplementary material for: Pre‐Existing Diabetes Alters Pulmonary Inflammatory Gene Expression Priming for Injury
Source: FASEB J. 2025 Jul 14;39(14):e70804. doi: 10.1096/fj.202500816R (PMC12257442; doi:10.1096/fj.202500816R)
Supplement: Supplementary file 1 — Appendix S1. [file FSB2-39-e70804-s001.zip › fsb270804-sup-0002-TableS1.docx]

**Supplemental Table 1. List of primes utilized in the analysis.**

| Gene name | Forward primer | Reverse primer |
| --- | --- | --- |
| IL-1β | CCAAGCAACGACAAAATACC | GTTGAAGACAAACCGTTTTTCC |
| IL-10 | GCTCTTACTGACTGGCATGAG | CGCAGCTCTAGGAGCATGTG |
| IL-6 | AGACAAAGCCAGAGTCCTTCAG | TGCCGAGTAGATCTCAAAGTGA |
| IL-4 | GGTCTCAACCCCCAGCTAGT | GCCGATGATCTCTCTCAAGTGAT |
| GAPDH | TGGTGAAGGTCGGTGTGAAC | CCATGTAGTTGAGGTCAATGAAGG |
| TNF-α | GGTCCCCAAAGGGATGAGAA | TGAGGGTCTGGGCCATAGAA |
| CXCL-1 | CTGGGATTCACCTCAAGAACATC | CAGGGTCAAGGCAAGCCTC |
